# ATP-Gated P2X7-Ion Channel on Kidney-Resident Natural Killer T Cells and Memory T Cells in Intrarenal Inflammation

**DOI:** 10.1681/ASN.0000000564

**Published:** 2024-12-09

**Authors:** Marten Junge, Nastassia Liaukouskaya, Nicole Schwarz, Carolina Pinto-Espinoza, Alessa Z. Schaffrath, Björn Rissiek, Christian F. Krebs, Guido Rattay, Hans-Willi Mittrücker, Nicola M. Tomas, Annette Nicke, Friedrich Haag, Tobias B. Huber, Catherine Meyer-Schwesinger, Friedrich Koch-Nolte, Nicola Wanner

**Affiliations:** 1 Institute of Immunology, University Medical Center Hamburg-Eppendorf, Hamburg, Germany; 2 III. Department of Medicine, University Medical Center Hamburg-Eppendorf, Hamburg, Germany; 3 Hamburg Center for Kidney Health (HCKH), University Medical Center Hamburg-Eppendorf, Hamburg, Germany; 4 Department of Neurology, University Medical Center Hamburg-Eppendorf, Hamburg, Germany; 5 Department of General, Visceral and Thoracic Surgery, University Medical Center Hamburg-Eppendorf, Hamburg, Germany; 6 Walther Straub Institute for Pharmacology and Toxicology, Ludwig-Maximilians-Universität München, Munich, Germany; 7 Institute of Cellular and Integrative Physiology, University Medical Center Hamburg-Eppendorf, Hamburg, Germany

**Keywords:** GN, glomerulus, immunology, immunology and pathology

## Abstract

**Key Points:**

- Parenchymal T cells in the kidney expressed much higher levels of P2X7 than vascular T cells.
- P2X7-blocking nanobodies uncover a large fraction of kidney-resident natural killer T and tissue-resident memory T cells.
- These cells were lost during cell preparation because of activation of P2X7 by NAD^+^ released from damaged cells, unless blocked by nanobodies.

**Background:**

The P2X7 ion channel, a key sensor of sterile inflammation, has been implicated as a therapeutic target in GN, and P2X7-antagonistic nanobodies can attenuate experimental GN. However, little is known about the expression of P2X7 on renal immune cells.

**Methods:**

We used conventional immunofluorescence of kidney sections and intraperitoneal injection of nanobodies in mice followed by flow cytometry analysis of parenchymal T cells and RNA sequencing to elucidate the expression and function of P2X7 on parenchymal and vascular immune cells in the mouse kidney.

**Results:**

Our study showed that parenchymal T cells, including a large subset of natural killer T cells and CD69^+^ tissue-resident memory T cells, display much higher cell surface levels of P2X7 than vascular T cells. After a single intraperitoneal injection of P2X7-blocking nanobodies, P2X7 on parenchymal T cells was fully occupied by the injected nanobodies within 30 minutes. This resulted in an effective protection of these cells from nicotinamide adenine dinucleotide–induced cell death during cell preparation. Conversely, systemic injection of nicotinamide adenine dinucleotide that mimics sterile inflammation results in the selective depletion of P2X7^hi^CD69^hi^ T cells from the kidney parenchyma.

**Conclusions:**

Our study uncovered a novel purinergic regulatory mechanism affecting kidney-resident T-cell populations.

## Visual Abstract

**Figure:**
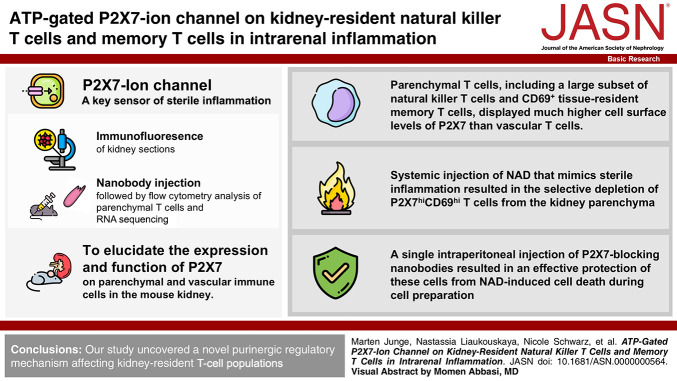

## Introduction

The P2X7 ion channel is a key sensor of sterile inflammation gated by high concentrations of extracellular ATP or by NAD-dependent ADP-ribosylation catalyzed by the ARTC2.2 ectoenzyme.^1–7^ P2X7 has been implicated as a therapeutic target in GN. Expression of P2X7 was upregulated during kidney inflammation.^6^ P2X7-deficient mice showed a milder course of antibody-mediated GN, and treatment of wild-type mice with small-molecule P2X7 antagonists reduced disease severity.^5,8,9^ Similarly, treatment of mice with P2X7-blocking nanobodies ameliorated antipodocyte GN, while P2X7-potentiating nanobodies enhanced inflammation.^10^ However, the expression and function of P2X7 by immune cells of the kidney are largely unknown.

Nanobodies correspond to the variable domain of heavy-chain antibodies that naturally occur in camelids.^11,12^ They show a remarkable capacity to bind to and block functional protein domains.^13^ Owing to their smaller size, they show better tissue penetration *in vivo* than conventional antibodies.^14^ As highly soluble protein domains, nanobodies can easily be fused to other proteins, including nanobodies of the same or a different specificity.^11,15^ In this study, we use *t*_1/2_-extended nanobody (Nb) dimers (half-life-extended [HLE] Nb-dimers fused to an albumin-specific Nb) to target P2X7 on the cell surface of kidney-resident T cells.^10^

The goal of our study was to assess the expression and function of P2X7 on parenchymal and vascular T cells in the kidney using P2X7-blocking nanobodies.

## Methods

### Animals

Wild-type, *P2rx7*^−/−^ (B6.129P2-P2rx7tm1Gab/J),^16^ and P2X7-enhanced green fluorescent protein (EGFP) (Tg[RP24-114E20-P2X7-His-StrepEGFP]Ani [line 17])^17^ C57BL/6 mice (8–12 weeks old) were bred in the animal facility of the University Medical Center Hamburg-Eppendorf. Animals had free access to water and standard animal chow. All experiments were performed with approval of the regulatory committee (Hamburger Behörde für Gesundheit und Verbraucherschutz, Veterinärwesen/Lebensmittelsicherheit, G12/130). All methods were performed in accordance with the Animal Research: Reporting of *In Vivo* Experiments guidelines and regulations.

### Recombinant Nanobodies

Recombinant HLE Nb 13A7-dimers and HLE Nb s+16a-dimers were produced in transiently transfected HEK293-6E cells and purified by immobilized metal affinity chromatography using Ni-nickel-nitrilotriacetic acid immobilized on agarose beads as previously described.^10,18,19^ Purified nanobodies were conjugated to AlexaFluor647 by random conjugation to amino groups according to the manufacturer's instructions (Molecular Probes).

### Systemic Injections of Nanobodies and NAD^+^

Purified nanobodies were adjusted to a concentration of 0.5 mg/ml in physiological saline solution and injected either intraperitoneally (i.p.) or intravenously into the tail vein at the indicated doses. NAD^*+*^ was adjusted to a concentration of 10 mg/ml in PBS, pH 7.0, and 200 *µ*l, *i.e*., 2 mg of nicotinamide adenine dinucleotide (NAD), were injected into the tail vein.^20^ Whole kidneys were used as tissue input. Bivalent HLE nanobodies bound to P2X7 on the cell surface were detected with mAb77, a mouse IgG1 mAb directed against Alb8 (Ablynx), followed by a BV421-conjugated mouse IgG1-specific secondary mAb (RMG1-1, BioLegend) (Supplemental Figure 1).

### Labeling of Vascular CD45^+^ Cells

For labeling of vascular CD45^+^ cells (CD45iv) and differentiation from tissue-resident parenchymal immune cells, peridinin chlorophyll protein–conjugated *α*-CD45 mAb (BioLegend, clone 30-F11) was adjusted to a concentration of 10 *µ*g/ml, and 100 *µ*l (1 *µ*g) were injected into the tail vein 3 minutes before sacrifice. Cell suspensions from kidneys were stained *ex vivo* with a panel of antibodies, including the same CD45-specific mAb conjugated to a different fluorochrome (AF680, AF700, or APC-Cy7) (CD45) as previously demonstrated.^21^

### Preparation of Primary Kidney Leukocytes

Kidneys were cut into small 1–5 mm fragments in ice-cold DMEM-containing 10% fetal calf serum and 0.4 mg/ml collagenase D (Roche) and 0.01 mg/ml desoxyribonuclease type 1 (Sigma). Tissue fragments were minced using a GentleMACS dissociator (Miltenyi Biotec) at 37°C for 30 minutes. Cell pellets were gently resuspended and layered onto a 37% Percoll gradient (GE healthcare, Uppsala, Sweden). Band corresponding to leukocytes was collected, and cells were washed in DMEM-10% fetal calf serum. Erythrocytes were lyzed with ammonium chloride potassium erythrocyte lysis buffer (155 mM NH_4_Cl, 10 mM KHCO_3_, 0.1 mM EDTA, pH 7.2). Cells were resuspended and filtered through a 40-*µ*m mesh. In some experiments, the cardiovascular system was perfused by intracardial injection of 20 ml of PBS heparin using a peristaltic pump (2 ml/min).

### Flow Cytometry

The following mAbs from BioLegend (BL) and BD Biosciences (BD) were used for flow cytometry: *α*-CD45 (30-F11, BL), *α*-CD4 (GK1.5 or RM4-5, BL), *α*-CD8 (53-6.7, BL), *α*-CD69 (clone H1.2F3, BL), *α*-CD11b (M1/70, BL), *α*-TNF-*α* (MP6-XT22, BD), and *α*-IFN-*γ* (XMG1.2, BD). The mAbs *α*-ARTC2.2 (Nika102) and *α*-P2X7 (RH23A44) were discovered in our laboratory.^18,22,23^ Phycoerythrin-labeled CD1d tetramer (CD1dtet, loaded with the analogue of *α*-galactosylceramide PBS-57) was kindly provided by the National Institutes of Health tetramer core facility. Dead-cell staining was performed using the live/dead Fixable Read Dead Stain Kit (Invitrogen). Data were collected using a BD fluorescence-activated cell sorting (FACS) Celesta or FACSymphony A1 Cell Analyzer and BD FACSDiva Software, and data were analyzed using FlowJo Software (Tree Star). For intracellular cytokine staining, cells were stimulated by incubation with phorbol-12-myristate-13-acetate (PMA) (50 ng/ml; Sigma-Aldrich) and ionomycin (1 *μ*g/ml; Calbiochem-Merck) in an *ex vivo* medium (Lonza) for 4 hours at 37°C, 5% CO_2_. After 30 minutes of incubation, brefeldin A (10 *μ*g/ml; Sigma-Aldrich) was added.

### Quantification of Cell Numbers

To quantify cells by flow cytometry, cell samples were prepared as described above. After the final wash, 50 *µ*l of Invitrogen/Thermo Fisher CountBright Absolute Counting Beads were added according to the manufacturer's instructions. The beads were identified by their emission in the 730-nm range after 640-nm excitation. Absolute cell concentration in the original suspension was calculated by comparing the ratio of beads to cells obtained from the flow cytometry data with the bead concentration provided by the manufacturer.

### Immunofluorescence Stainings

Primary antibodies: mouse IgG2 *α*-green fluorescent protein (GFP) B2 (1:100, sc-9996; Santa Cruz Biotechnology), rabbit *α*-CD3 (1:10, ab 135372; Abcam), rabbit *α*-CD4 (1:100, ab 183685; Abcam), rat *α*-Endomucin (1:400, sc-65495; Santa Cruz Biotechnology), rabbit *α*-Iba1 (1:250, 019-19741; Wako Chemicals), rhodamine-labeled wheat germ agglutinin (1:400, wheat germ agglutinin, Vector, RL-1022), and *Lotus tetragonolobus* lectin (FL-1321-2, Vector Laboratories).^24^ Secondary antibodies and Hoechst 33342 (H3570) were obtained from Invitrogen.

Kidneys of healthy adult mice were perfused with PBS and 4% paraformaldehyde (PFA) through the *arteria renalis* and fixed in 4% PFA overnight, dehydrated, and embedded in paraffin. The embedded tissue was sectioned at 3 *μ*m with a Leica Microtome. The sections were deparaffinized in xylol and rehydrated in an ethanol series, followed by washing in PBS. Heat-mediated antigen retrieval was performed for 30 minutes using Dako buffer pH 9 in a steamer. The sections were blocked with PBS containing 5% bovine serum albumin and incubated for 1 hour with primary antibodies. After three PBS rinses, AlexaFluor-conjugated secondary antibodies were applied for 1 hour. Microscopy and acquisition of images were performed using a LSM800 with Airyscan 1 for high-resolution confocal microscopy using ZENblue software or a Leica thunder microscope.

For detection of systemically injected AlexaFluor647-conjugated HLE Nb 13A7-dimers, kidney samples were prepared 60 minutes after Nb injection and frozen in liquid nitrogen in an *N*-butan (Sigma) bath. Frozen sections (5 *µ*m thick) were air-dried and fixed with 4% PFA for 8 minutes at room temperature and washed with PBS. Unspecific binding was blocked (5% horse serum, 0.05% Triton X-100 in PBS, 30 minutes at room temperature). Staining was evaluated with the laser scanning microscope 510 META using the laser scanning microscope Image Browser Software (Zeiss, Jena, Germany).

### RNA Sequencing

T-cell subpopulations (vascular CD4^+^, CD1dtet^+^/CD69^+^ parenchymal natural killer T cells, CD1dtet^−^/CD4^+^/CD69^+^ tissue-resident memory [TRM] T cells and CD1dtet^−^/CD69^−^ non-TRM T cells) were sorted, and total RNA was extracted using the Qiagen RNeasy Micro Kit. RNA sequencing was performed by the Leibniz Institute of Virology, Hamburg. Quality control was performed with FastQC (Babraham Bioinformatics). Raw reads were trimmed using Trim Galore! (Babraham Bioinformatics) and mapped with RNA Star to mm9 using Galaxy Freiburg. Read counts were extracted with htseq-count; rlog transformation was performed using DESeq2.^25^ For preranked gene set enrichment analysis, fast gene set enrichment analysis was used.^26^ RNA-sequencing raw data were deposited in the Gene Expression Omnibus (GEO) of the National Center for Biotechnology Information accessible through GEO Series accession number GSE241191 (https://www.ncbi.nlm.nih.gov/geo/query/acc.cgi?acc=GSE241191).

### Statistical Analyses

Statistical analyses were performed with GraphPad PRISM9 software. One-sided Mann–Whitney *U* test was used for comparison between two groups.

## Results

### Immunofluorescent Analyses Showed P2X7 Expression by Kidney-Resident Cells

**Figure 1:**
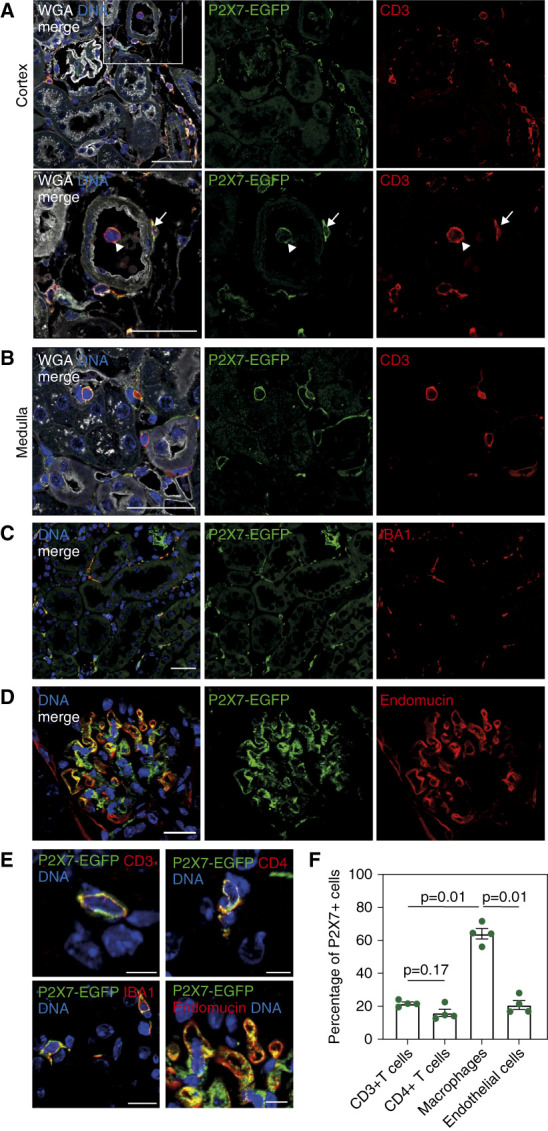
**Immunofluorescent analyses show P2X7 expression by kidney-resident cells.** (A) Kidney cortex area of a P2X7-EGFP transgenic mouse stained with WGA and antibodies specific for GFP and CD3. Arrows mark a parenchymal T cell and arrowheads a vascular T cell. Scale bar: 50 *µ*m. (B) Kidney medulla of a P2X7-EGFP transgenic mouse stained with WGA and antibodies specific for GFP and CD3. Scale bar: 50 *µ*m. (C) Kidney sections were stained with *α*-GFP (green) and *α*-IBA1 (red) antibodies for detection of coexpression of P2X7-EGFP with kidney MΦ. Scale bar: 20 *μ*m. (D) Glomerular staining of anti-GFP (green) and anti-Endomucin (red) antibodies for detection of coexpression of P2X7-EGFP with endothelial cells. Scale bar: 30 *μ*m. (E) Zoomed-in stainings of anti-GFP (green) with anti-CD3, anti-CD4, anti-IBA1, and anti-Endomucin (red) antibodies for detection of coexpression of P2X7-EGFP. Scale bar: 10 *μ*m (upper panels) and 20 *µ*m (lower panels). (F) Percentage of P2X7^+^ cells coexpressing CD3, CD4, IBA1, and Endomucin. Data represent mean±SEM, *n*=4. EGFP, enhanced green fluorescent protein; GFP, green fluorescent protein; WGA, wheat germ agglutinin.

We previously showed that HLE Nb-dimers of the P2X7-blocking Nb 13A7 (HLE Nb 13A7-dimer) ameliorate inflammation in a mouse model of antibody-induced GN.^10^ To better understand the role of P2X7 for kidney-resident cells, we analyzed histological sections of our recently developed P2X7-EGFP transgenic mice, which overexpress a P2X7-EGFP fusion protein from a bacterial artificial chromosome encompassing the entire P2X7 gene locus.^17^ Immunofluorescent staining of kidney sections revealed P2X7 expression mostly on resident immune cells: Costaining of markers for T cells and macrophages (MΦ) with a GFP-specific antibody confirmed the expression of P2X7 by interstitial CD3^+^ T cells (Figure 1, A and B) and IBA^+^ MΦ (Figure 1C). Glomerular staining showed costaining with endothelial marker Endomucin (Figure 1D). Quantification of cell types in histological sections showed that MΦ comprised the majority (approximately 60%) of P2X7-positive cells; approximately 20% of P2X7-positive cells were CD3^+^ (including 16% CD4^+^ cells) and another approximately 20% were positive for Endomucin (Figure 1, E and F).

### Renal Parenchymal T Cells Expressed Much Higher Levels of P2X7 Than Their Vascular Counterparts

**Figure 2:**
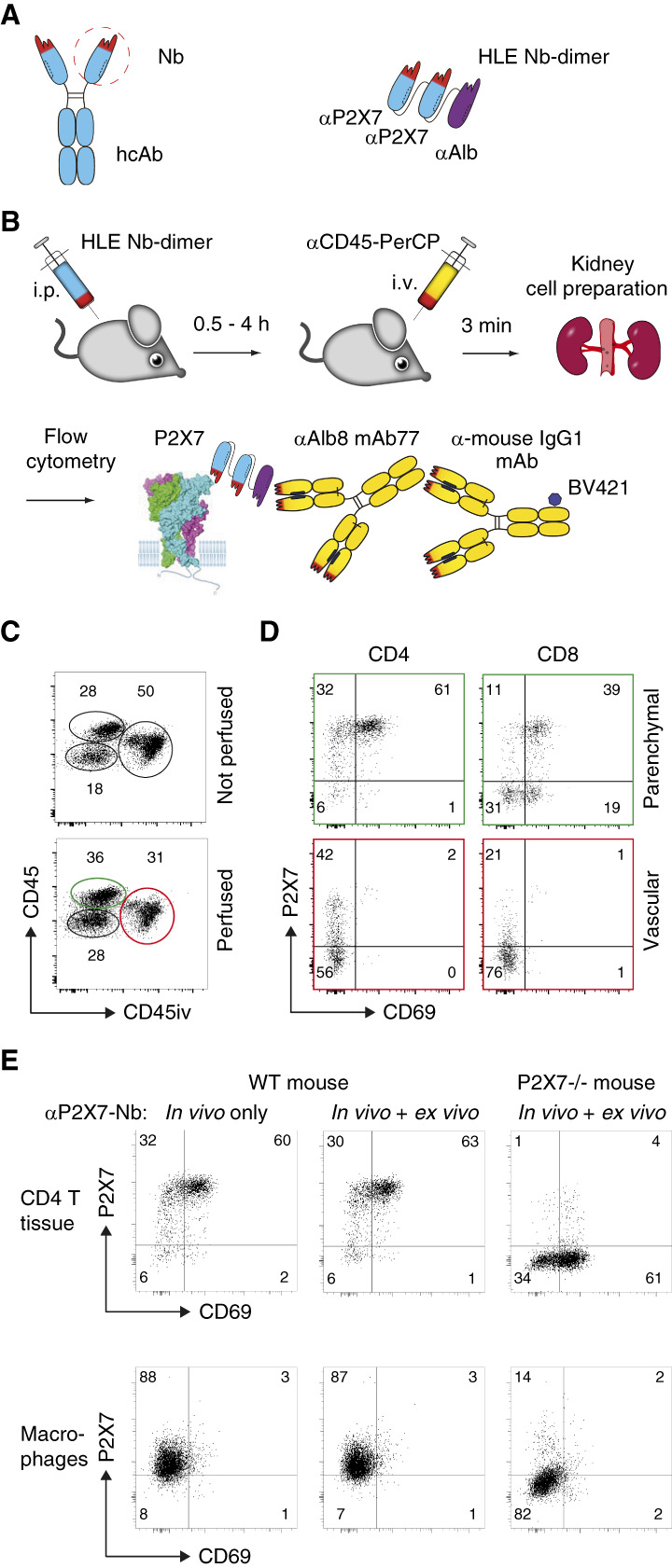
**Renal parenchymal T cells express much higher levels of P2X7 than their vascular counterparts.** (A) A *t*_1/2_-extended Nb-dimer (HLE Nb-dimer) was generated by fusing a dimer of the P2X7-antagonistic Nb 13A7 to the albumin-specific Nb Alb8 using a flexible Glycine–Serine linker. (B) The HLE Nb-dimer was injected i.p. 0.5–4 hours before sacrifice. To specifically stain intravascular leukocytes, PerCP-conjugated *α*-CD45 mAb was injected i.v. 3 minutes before sacrifice. Leukocytes were purified from dissociated kidneys by density gradient centrifugation and analyzed by flow cytometry. Bound HLE Nb-dimers were detected using Alb8-specific mAb77 (mouse IgG1), followed by BV421-conjugated mouse IgG1-specific mAb. (C) Vascular CD45^+^ leukocytes (red circle) can be distinguished from parenchymal CD45^+^ leukocytes (green oval) by the previous i.v. injection of conjugated *α*-CD45 mAb before sacrifice. Perfusion of the kidneys with PBS results only in partial reduction of vascular cells. (D) Specific detection of systemically injected HLE Nb-dimer on the surface of parenchymal (top) and vascular (bottom) CD4^+^ and CD8^+^ T cells. (E) Addition of the HLE Nb-dimer *ex vivo* did not result in any detectable increase of staining intensity. Cells from P2X7^−/−^ mice injected with the HLE Nb-dimer were used as specificity controls. hcAb, heavy-chain antibody; HLE Nb, half-life-extended nanobody; i.p., intraperitoneally; i.v., intravenously; Nb, nanobody; PerCP, peridinin chlorophyll protein; WT, wild-type.

To further determine the therapeutic potential of P2X7 antagonists in kidney disease, we evaluated the P2X7-expressing kidney-resident T-cell populations. For this, we developed a sensitive flow cytometry assay to detect systemically injected HLE Nb 13A7-dimers on the surface of renal T cells *ex vivo* (Figure 2, A and B). Injection of a fluorochrome-conjugated CD45-specific mAb shortly before sacrifice allowed us to distinguish vascular from parenchymal leukocytes (Figure 2C) in wild-type mice. Perfusion of the vasculature with PBS effectively removed erythrocytes, as evidenced by macroscopic pallor of the kidney. However, a substantial fraction (often the majority) of vascular leukocytes was still recovered from perfused kidneys (Figure 2C, lower panel).

In the parenchymal compartment, two distinct subsets of leukocytes could be delineated on the basis of CD45 expression levels. The CD45^hi^ subset (approximately 60% of parenchymal cells) consisted mainly of lymphocytes, and the CD45^int^ subset contained mainly MΦ. Flow cytometry analysis confirmed the results of the immunofluorescence staining, revealing significantly elevated cell surface levels of P2X7 in most of the parenchymal CD4^+^ and CD8^+^ T-cell subsets compared with their vascular counterparts (Figure 2D). Consistently, most of the parenchymal T cells, but not of vascular T cells, expressed CD69, a marker of tissue residency.^27^ In both CD4^+^ and CD8^+^ parenchymal T-cell subsets, only a fraction of cells (approximately 5% of CD4^+^ cells, approximately 30% of CD8^+^ cells) displayed low or undetectable levels of P2X7 and CD69.

Staining of cell surface–bound HLE Nb 13A7-dimer using mAb77 revealed essentially complete occupancy of P2X7 on parenchymal T cells within 60 minutes after injection of a therapeutic dose (approximately 2 mg/kg) (Figure 2E). Incubation of renal cells with additional HLE Nb 13A7-dimer *ex vivo* did not further increase the staining intensity of T cells, confirming that P2X7 on parenchymal T cells was fully covered by the injected nanobodies (Figure 2E). By contrast, cells recovered from HLE Nb 13A7-dimer–injected P2X7-deficient mice did not show any signal, confirming the specificity of the staining strategy (Figure 2E). After the injection of Alexa647-conjugated HLE Nb 13A7-dimer, we could detect the labeled HLE Nb-dimer on the cell surface of tissue-resident cells in frozen sections (Supplemental Figure 2).

### *t*_1/2_-Extended P2X7-Specific Nb-Dimers Fully Covered P2X7 on Renal Parenchymal CD4^+^ Cells within 10 Minutes after Intraperitoneal Injection

To determine the minimal dose and time required for effective targeting of P2X7 on renal T cells, we performed dose titration and kinetic analyses (Supplemental Figure 3). To assess the dose of nanobodies required to fully cover parenchymal T cells, we injected titrated doses of nanobodies and analyzed occupancy of P2X7 by the injected nanobodies on vascular and parenchymal kidney cells 60 minutes after injection. The results revealed that a dose of 10 *µ*g (approximately 0.5 mg/kg) sufficed to completely cover P2X7 on parenchymal T cells (Supplemental Figure 3A). The kinetic analyses showed that P2X7 was already fully covered on vascular and parenchymal T cells in the kidney within 3 minutes after i.p. injection of 10 *µ*g of HLE Nb 13A7-dimer, with persisting coverage after 10 and 30 minutes (Supplemental Figure 3B).

### P2X7-Blocking Nanobodies Uncovered a Large Fraction of Kidney-Resident Natural Killer T Cells

**Figure 3:**
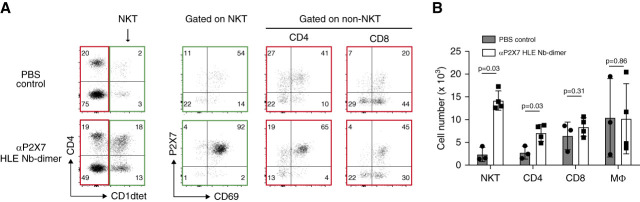
**P2X7-blocking nanobodies uncover a large fraction of kidney-resident natural killer T cells.** (A) Mice were injected i.p. with the P2X7-specific *t*_1/2_-extended Nb dimer (HLE Nb-dimer) and with fluorochrome-conjugated CD45-specific mAb 4 hours and 3 minutes before sacrifice, respectively, and kidney leukocytes were analyzed by flow cytometry as in Figure 2. Gating was performed sequentially on parenchymal lymphocytes (CD45iv^−^, CD45^high^) and on parenchymal CD1dtet-positive natural killer T cells, as well as on non–natural killer T cells (CD1dtet^−^), followed by gating on CD4^+^ and CD8^+^ T cells. (B) Absolute numbers of cells recovered from mice injected with PBS only (*n*=3) and from mice injected with the HLE Nb-dimer (*n*=4). Error bars indicate SD. MΦ, macrophage; NKT, natural killer T cells.

Comparative analyses of kidney T cells recovered from mice that received an injection of the P2X7-blocking HLE Nb 13A7-dimer with control mice injected with PBS revealed striking differences in the composition and phenotype of parenchymal T-cell subsets, particularly in the proportion of natural killer T cells (Figure 3 and Supplemental Figures 4 and 5). Tissue-resident natural killer T cells were previously reported to highly express P2X7 in the liver.^19,28,29^ They express unique T-cell receptors that recognize carbohydrate antigens presented by the major histocompatibility complex class I–related molecule CD1d.^30^ They can be distinguished from classic T cells by staining with fluorochrome-conjugated CD1dtet loaded with *α*-galactosylceramide binding specifically to the invariant TCR on invariant natural killer T cells.^31^ Much higher proportions of natural killer T cells were recovered from mice injected with HLE Nb 13A7-dimer than from controls (31% versus 5%) (Figure 3A). Notably, natural killer T cells constituted a major subset (30%–50%) of parenchymal T cells, but only a marginal subset of intravascular T cells (<1%) (not shown). Most (>90%) of the parenchymal kidney natural killer T cells coexpressed high levels of P2X7 and CD69 (Figure 3A). Approximately 60% of these cells coexpressed CD4, the coreceptor of the T-cell receptor binding to major histocompatibility complex class II. Higher proportions of parenchymal P2X7^hi^CD69^hi^ cells were also recovered in the CD4^+^ and CD8^+^ T-cell subsets from mice that had been treated with the P2X7-blocking HLE Nb-dimer compared with the control mice (Supplemental Figure 6). These observations suggest that P2X7^hi^CD69^hi^ cells might be lost during cell preparation due to P2X7-mediated cell death and, conversely, that the injected P2X7-antagonistic HLE Nb-dimer protected these cells from nucleotide-induced death during cell preparation. Quantification of cell numbers by the addition of fluorescently labeled beads to our samples allowed us to estimate the recovered number of renal parenchymal T cells out of the control mice (4000–5000 T cells) and the HLE Nb-dimer–treated mice (20,000–25,000 T cells), corresponding to approximately five-fold more T cells per nephron in the treated mice (Figure 3B).

### Parenchymal Renal T Cells Coexpressed High Levels of P2X7 and ARTC2.2

**Figure 4:**
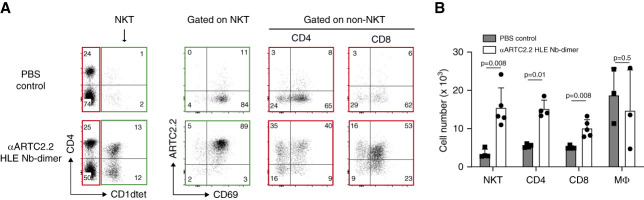
**ARTC2.2-blocking nanobodies confirm large subsets of CD69**^**+**^ **kidney-resident T cells.** (A) Mice were injected with PBS only or with the *t*_1/2_-extended Nb-dimer (HLA-Nb-dimer) of the ARTC2.2-blocking Nb s+16a, and with the fluorochrome-conjugated *α*-CD45 60 and 3 minutes before sacrifice, respectively, and kidney leukocytes were analyzed by flow cytometry as in Figure 2. Gating was performed sequentially on parenchymal lymphocytes (CD45iv^−^, CD45^high^) and on parenchymal CD1dtet-positive natural killer T cells, as well as on non–natural killer T cells (CD1dtet^−^), followed by gating on CD4^+^ and CD8^+^ T cells. (B) Absolute numbers of cells recovered from mice injected with PBS (*n*=3) and from mice injected with ARTC2.2-blocking HLE Nb-dimer (*n*=3–5). Error bars indicate SD.

We previously observed that NAD^+^ released during cell stress activates P2X7 on cells coexpressing P2X7 and ARTC2.2, a toxin-related ectoenzyme that catalyzes ADP-ribosylation of P2X7 at Arg125 near the ATP-binding pocket.^1,19,20,32–35^ We therefore analyzed whether ARTC2.2 is coexpressed by parenchymal kidney natural killer T cells and whether ARTC2.2 can be reached and blocked on these cells by the ARTC2.2-antagonistic Nb s+16a.^18,19,29,36,37^ To this end, we used the flow cytometry assay described in Figure 2 to monitor targeting of ARTC2.2 on kidney-resident T cells by i.p. injected HLE Nb-dimers of the ARTC2.2-antagonistic Nb s+16a (HLE Nb s+16a-dimer, Figure 4). The results showed that kidney-resident natural killer T cells indeed coexpress high levels of ARTC2.2 and P2X7 (Figure 4A). Moreover—similar to P2X7—ARTC2.2 is reached and fully covered by nanobodies within 60 minutes after i.p. injection. Similar to the treatment with the P2X7-antagonistic HLE Nb 13A7-dimer, treatment with the ARTC2.2-blocking HLE Nb s+16a-dimer resulted in the recovery of a large proportion of parenchymal natural killer T cells as well as of P2X7^hi^CD69^hi^ subpopulations of parenchymal CD4^+^ and CD8^+^ T cells (Figure 4). These results suggest that NAD^+^ released during cell preparation compromised the viability of kidney-resident natural killer T cells and that blockade of ARTC2.2-catalyzed ADP-ribosylation during cell preparation protected these cells from NAD-induced cell death.

### Mimicking Sterile Inflammation by Systemically Injecting NAD^+^ Resulted in the Selective Depletion of Kidney-Resident Natural Killer T Cells and P2X7^hi^CD69^hi^ CD4^+^ T Cells

**Figure 5:**
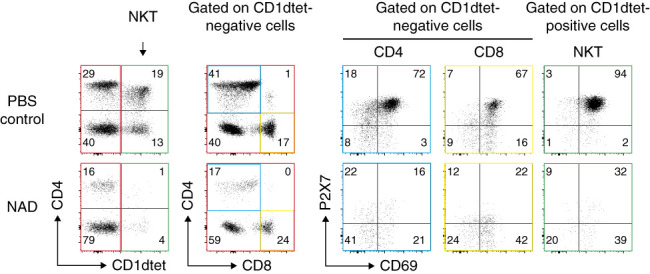
**Mimicking sterile inflammation by systemically injecting NAD**^**+**^ **results in the selective depletion of kidney-resident natural killer T cells and P2X7**^**hi**^**CD69**^**hi**^ **CD4**^**+**^ **T cells.** Mice received intravenous injections of NAD or PBS 4 hours before sacrifice. To block ARTC2.2-catalyzed ADP-ribosylation of P2X7 during cell preparation, mice received an intraperitoneal injection of the *t*_1/2_-extended Nb dimer (HLE Nb-dimer) of the ARTC2.2-blocking Nb s+16a 60 minutes before sacrifice. PerCP-conjugated *α*-CD45 mAb was injected 3 minutes before sacrifice, and kidney leukocytes were prepared from whole kidneys, stained, and analyzed by flow cytometry as in Figure 4. Gating was performed on parenchymal lymphocyte subsets (CD45^hi^). NAD, nicotinamide adenine dinucleotide.

The evident ARTC2.2- and P2X7-dependent loss of natural killer T cells during cell preparation suggests that these cells may also be lost in response to NAD^+^ released during sterile inflammation. To mimic sterile inflammation,^20^ we intravenously injected NAD^+^ and analyzed the composition and phenotype of T-cell subpopulations recovered from these mice 24 hours after NAD^+^ injection (Figure 5 and Supplemental Figure 7). To block NAD^+^-dependent ADP-ribosylation of P2X7 in response to NAD^+^ released during cell preparation, both NAD^+^- and PBS-treated mice received injections of the ARTC2.2-blocking HLE Nb s+16a-dimer 60 minutes before analysis. The results showed that systemic injection of NAD^+^ resulted in the preferential depletion of kidney-resident natural killer T cells. Similarly, NAD^+^ injection also resulted in a substantial depletion of P2X7^hi^CD69^hi^ CD4^+^ and CD8^+^ T cells.

### Renal Parenchymal T Cells Express Memory T-Cell Markers

**Figure 6:**
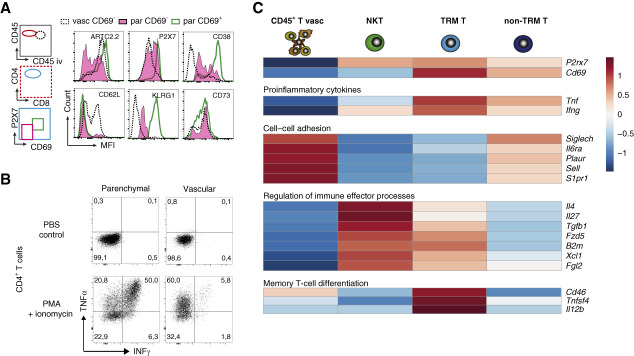
**Renal parenchymal T cells express memory cell–like markers.** (A) Mice received an intraperitoneal injection of *t*_1/2_-extended Nb-dimer (HLE Nb-dimer) of the ARTC2.2-blocking Nb s+16a 60 minutes before sacrifice. Intravascular staining with PerCP-conjugated *α*CD45 mAb was performed 3 minutes before sacrifice. Leukocytes were purified from dissociated kidneys by density gradient centrifugation, stained with a panel of fluorochrome-conjugated antibodies, and analyzed by flow cytometry. Gating was performed on vascular (vasc) and parenchymal (par) CD4^+^ T cells (CD1dtet^−^). (B) The ARTC2.2-blocking HLE Nb-dimer and PerCP-conjugated *α*-CD45 mAb were injected i.p. 60 minutes and i.v. 3 minutes before sacrifice, respectively. Kidney leukocytes were purified and incubated for 4 hours in the absence or presence of phorbol myristic acid, the calcium ionophore ionomycin, and the Golgi inhibitor brefeldin A. After staining with AF700-conjugated *α*-CD45 and *α*-CD4, cells were fixed and permeabilized. Cells were further stained with TNF-*α*– and INF-*γ*–specific antibodies before analysis by flow cytometry. Gating was performed on vascular and parenchymal CD4^+^ T cells. (C) RNA sequencing of vascular (vasc) CD45^+^ T, natural killer T, CD4^+^ CD69^+^ TRM T, and CD4^+^CD69^−^ non-TRM T-cell populations shows differences in gene expression of proinflammatory genes, cell–cell adhesion molecules, regulators of immune effector processes, and memory T cell differentiation. Colors represent z-score of regularized log2 transformation. PMA, phorbol-12-myristate-13-acetate; TRM, tissue-resident memory.

We next sought to assess other cell surface markers of the P2X7^+^ renal T-cell populations, such as CD38, a cyclic ADP ribose hydrolase regulating the availability of NAD^+^ as a substrate for ARTC2.2^38^; CD73, an ecto-5-nucleotidase hydrolyzing AMP to adenosine; cell adhesion molecule CD62L (L-selectin/Sell) mediating adhesion of vascular leukocyte–endothelial cell interactions; and killer cell lectin-like receptor subfamily G member 1, a ligand for cadherins considered to be a marker of senescent T cells (Figure 6A). Next to expression of P2X7 and ARTC2.2, CD38 was relatively high in CD69^+^P2X7^+^ cells, whereas CD62L expression was low on CD69^+^ and CD69^−^ parenchymal cells, compared with vascular cells (Figure 6A). While the expression of killer cell lectin-like receptor subfamily G member 1 was also relatively high in CD69^+^P2X7^+^ cells, CD73 showed intermediate expression in all measured cell populations (Figure 6A). To assess the functional states of CD69^+^P2X7^+^ cells, we examined the capacity of parenchymal and vascular T cells to express the potent proinflammatory cytokines TNF-*α* and INF-*γ* in response to a 4-hour treatment with PMA (Figure 6B). The results revealed that parenchymal kidney T cells expressed much higher levels of these cytokines in response to PMA treatment than their vascular counterparts.

To gain further information on gene expression in the different cell types, bulk RNA sequencing of sorted vascular CD4^+^ T cells, parenchymal natural killer T cells, and CD69^+^ TRM T cells and CD4^+^CD69^−^ cells was performed (Supplemental Figure 8). Normalized gene expression confirmed expression of *P2rx7* by parenchymal natural killer T and CD69^+^ T-cell population and of *Cd69* in the latter (Figure 6C). CD69^+^ TRM T cells showed the highest expression of proinflammatory cytokines *Tnf* and *Ifng* (Figure 6C). Within the top 50 regulated genes (Supplemental Figure 9), both natural killer T cells and TRM T cell subsets showed decreased expression of adhesion markers, such as *Cd62l* (*Sell*), *Plaur*, *Siglech*, and *S1pr1*, while expression of markers for immune effector processes, such as *Il4*, *Tgfb1*, *Xcl1*, and *Fgl2*, were increased (Figure 6C). Gene set enrichment analysis of the cell populations revealed increased translational activity and oxidative phosphorylation in CD69^+^ TRM T cells compared with CD4^+^ CD69^−^ (non-TRM T) cells, as well as in natural killer T cells compared with vascular T cells (Supplemental Figure 10), indicating changes in metabolism of P2X7^+^ natural killer T cells and TRM T-cell subsets. Finally, CD69^+^ T cells also showed increased expression of genes involved in memory T-cell differentiation, such as *Cd46*, *Tnfsf4*, and *Il12b* (Figure 6C).

## Discussion

The P2X7 ion channel is a potential therapeutic target in GN, yet little is known about its role in the kidney. In this study, we uncovered a large hitherto unknown subpopulation of parenchymal kidney-resident natural killer T cells that uniformly coexpress high levels of P2X7, CD69, and ARTC2.2. In stark contrast, the renal vascular T-cell compartment contained only a tiny subpopulation of natural killer T cells. Moreover, our results showed that a large fraction of parenchymal CD4^+^ T cells and a substantial fraction of parenchymal CD8^+^ T cells similarly coexpress high levels of P2X7, CD69, and ARTC2.2. Again, in striking contrast, the vascular compartment contained few, if any, T cells of this phenotype. Although the occurrence of P2X7^+^ TRM T cells was shown in the liver and P2X7 was shown to regulate natural killer T cells during autoimmune hepatitis,^28,32^ this is the first description of these cell subpopulations in the kidney.

Kinetic analysis of potentially therapeutic P2X7-specific HLE Nb-dimers injected *in vivo* showed a fast and efficient coverage of cell surface P2X7 molecules in the tissue.^10^ Furthermore, HLE Nb-dimer–mediated blockade of P2X7 or of the P2X7-activating ARTC2.2 ADP-ribosyltransferase had a strong effect on cell recovery: Without P2X7 blockage, parenchymal kidney T cells were highly sensitive to P2X7-mediated cell death. Using standard procedures for harvesting kidney leukocytes, the viability of these cells was severely compromised. Only few, if any, vital P2X7-expressing TRM T or natural killer T cells could be recovered from primary kidney cell suspensions. P2X7-mediated loss of parenchymal kidney T cells could be effectively prevented by a single intraperitoneal injection of either P2X7-blocking or ARTC2.2-blocking HLE Nb-dimers 60 minutes before sacrifice.^10,19^ These results imply that previous studies on kidney leukocytes warrant reinterpretation as standard tissue and cell isolation techniques likely led to loss of parenchymal kidney T cells by inadvertent release of NAD^+^.^39^ Previously reported disparities in cell yields resulting from cell preparation procedures have been documented, potentially causing an underestimation of specific cell types, such as memory CD8^+^ T cells in nonlymphoid tissues.^40^

Our findings cannot be translated directly to the human kidney because the human *ARTC2.2* gene is nonfunctional.^41,42^ However, a recent study found that human *ARTC1* (*ART1*), *i.e*., the closest paralogue of *ARTC2.2* in humans, can ADP-ribosylate P2X7 on human CD8^+^ T cells and thereby mediate NAD-induced cell death of these cells in the tumor microenvironment.^43,44^ It remains to be determined whether a similar mechanism operates also during human kidney inflammation.

Our results are in line with those of other recent studies that indicate upregulated expression of P2X7 as a hallmark of T-cell residency in a variety of tissues, including the gut, liver, and female reproductive tract.^32,45–47^ Furthermore, CD69, originally identified as a T-cell activation antigen, is now recognized as a marker of tissue residency.^48,49^ A recent comparative transcriptome analysis of sorted splenic tissue-resident memory cells (CD62L^lo^CD69^+^) and central memory cells (CD62L^hi^CD69^−^) identified *P2rx7* as the most highly upregulated gene.^46^ The results of our gene expression analysis also indicate that expression of *Cd69* and *P2rx7* by renal parenchymal CD4^+^ T cells justified classifying them as TRM T cells. By contrast, the P2X7^lo^ and P2X7^−^ T-cell populations showed lower expression of *Cd69* and higher expression of leukocyte cell adhesion factors, such as *Cd62l*. Shared factors expressed in both natural killer T cells and TRM T-cell subsets point to functional similarities, such as usage of similar cell chemotaxis mechanisms. Both cell subsets are highly susceptible to NAD^+^, indicating a highly alert state in the kidney parenchyma: Our results suggest that high expression of *P2rx7* renders tissue-resident T cells sensitive to nucleotides released during sterile inflammation. Indeed, systemic injection of NAD^+^ resulted in the selective depletion of P2X7^hi^CD69^+^ T cells and natural killer T cells from the kidney parenchyma.

In conclusion, our study has uncovered a novel P2X7-mediated regulatory mechanism for alerting parenchymal T cells in the kidney to the nucleotides released during sterile inflammation. This mechanism accounts for a severe compromise in the viability of parenchymal T cells during routine preparation of kidney cells. Preventing the activation of P2X7 during cell preparation, for instance, by systemic injection of P2X7-blocking or ARTC2.2-blocking HLE Nb-dimers, permits the recovery of viable parenchymal kidney T cells.

## Data Availability

Data related to transcriptomic, proteomic, or metabolomic data. Original data created for the study are available in a persistent repository. Experimental Data. GEO. https://www.ncbi.nlm.nih.gov/geo/query/acc.cgi?acc=GSE241191.

## Supplemental Material

Supplemental Figure 1. Fluorescence minus one control.

Supplemental Figure 2. Alexa647-conjugated anti-P2X7 nanobody staining of a parenchymal cell in the kidney.

Supplemental Figure 3. *t*_1/2_-extended P2X7-specific nanobody dimers fully cover P2X7 on renal parenchymal CD4^+^ cells within 10 minutes after intraperitoneal injection.

Supplemental Figure 4. Gating strategy and IgG control.

Supplemental Figure 5. Persistence of cell surface P2X7-Nbs.

Supplemental Figure 6. Treatment with P2X7-blocking HLE Nb-dimer leads to recovery of higher proportions of parenchymal P2X7^hi^CD69^hi^ cells in the CD4^+^ and CD8^+^ T-cell subsets.

Supplemental Figure 7. Systemic injection of NAD^+^ results in the selective depletion of kidney-resident CD1dtet^+^ NKT cells and CD4^+^ T cells.

Supplemental Figure 8. Gating strategy to isolate cell subsets by FACS sorting.

Supplemental Figure 9. Bulk RNA-sequencing analysis of FACS-sorted cell subsets.

Supplemental Figure 10. Gene set enrichment analysis between isolated cell subsets.
